# Chromoendoscopy with a Standard-Resolution Colonoscope for Evaluation of Rectal Aberrant Crypt Foci

**DOI:** 10.1371/journal.pone.0148286

**Published:** 2016-02-17

**Authors:** Marek Kowalczyk, Piotr Siermontowski, Dariusz Mucha, Tadeusz Ambroży, Marcin Orłowski, Krzysztof Zinkiewicz, Waldemar Kurpiewski, Krzysztof Paśnik, Iwona Kowalczyk, Agnieszka Pedrycz

**Affiliations:** 1 Department of Oncologic and General Surgery, University Hospital in Olsztyn, Poland; 2 Department of Maritime & Hyperbaric Medicine Department, Military Institute of Medicine Gdynia, Poland; 3 Academy of Physical Education in Cracow, Department of Physical Education and Sport, Cracow, Poland; 4 Centre for Diagnosis and Treatment of Gastrointestinal Diseases, Gdańsk, Poland; 5 2nd Department of General, Gastroenterologic and Gastrointestinal Oncologic Surgery, Medical University of Lublin, University Hospital No.1, Lublin, Poland; 6 Department of General, Oncologic, Metabolic and Thoracic Surgery, Military Institute of Medicine, Military Hospital in Warsaw, Poland; 7 Unit for Laboratory Diagnostics, University Hospital in Olsztyn, Poland; 8 Department of Histology and Embryology with Unit of Experimental Cytology, Medical University of Lublin, Poland; Rutgers, the State Univesity of New Jersey, UNITED STATES

## Abstract

Colorectal cancer (CRC) is the second most common cause of death worldwide. According to the theory by Vogelstein, colorectal carcinogenesis involves a series of successive changes in the normal colonic mucosa, starting with excessive proliferation and focal disorders of intestinal crypts, followed by adenoma and its subsequent malignant transformation. The first identifiable changes in CRC carcinogenesis are aberrant crypt foci (ACF). ACF are invisible during routine colonoscopy yet are well identifiable in chromoendoscopy using methylene blue or indigo carmine. High-resolution colonoscopes are used for assessment of ACF. The aim of the present study was to evaluate the usefulness of standard-resolution colonoscopy for identification of rectal ACF. The following parameters were evaluated: duration of chromoendoscopy of a given rectal segment, type of ACF, sensitivity and specificity of endoscopy combined with histopathological evaluation. The mean duration of colonoscopy and chromoendoscopy was 26.8 min. In the study population, typical ACF were found in 73 patients (p = 0.489), hyperplastic ACF in 49 (p = 0.328), and dysplastic ACF in 16 patients (p = 0.107). Mixed ACF were observed in 11 individuals (p = 0.073). The sensitivity of the method was found to be 0.96 whereas its specificity 0.99.

Identification of rectal ACF using standard-resolution colonoscopy combined with rectal mucosa staining with 0.25% methylene blue is characterised by high sensitivity and specificity.

## Introduction

Despite early detection screening programmes and modern diagnostic methods, colorectal cancer is still the second leading cause of death worldwide [1]. The identification of its potential precursors enables the removal of early lesions and implementation of appropriate preventive measures. According to the theory by Vogelstein, carcinogenesis of colorectal cancer involves a series of successive changes in the normal mucosa starting with excessive proliferation and focal disorders of intestinal crypts, through the development of adenoma to its malignant transformations [2]. Samowitz and colleagues have shown that mutations in the three “classical” genes of the Fearon-Vogelstein model, i.e. ACS, KRAS and P53, are observed only in about 10% of colorectal cancer cases [3]. Hanahan et al. have demonstrated that several alterations in metabolism and physiology of cells are required to develop a fully malignant phenotype [4].

During carcinogenesis, the cell acquires certain features that condition its expansive development:

1. ability to divide without the involvement of stimulating signals, e.g. activation of some oncogenes (RAS, c-myc)
2. ability to grow despite the action of inhibiting factors (loss of activity of suppressor genes, e.g. APC, p53, Smad2, Smad4, DCC, MCC
3. impaired apoptosis (e.g. production of insulin-like growth factor -IGF)
4. angiogenesis within a neoplastic tumour (production of VEGF)
5. invasiveness and capacity to metastasize (e.g. inactivation of E-cadherin, activation of telomerase).

The presence of such cells is not however tantamount with their ability to develop neoplasia. The essential features necessary for neoplasm proliferation, especially of solid tumours, include vascularisation due to induction of blood vessels, spread attributable to proteolytic activity (invasive growth) and penetration of lymphatic and blood vessels (formation of metastases). A relevant precondition for neoplastic growth is the failure to recognise the neoplastic cells by the immune system (an escape from the immune supervision).

The first identifiable changes in the carcinogenesis of colorectal cancer include aberrant crypt foci (ACF). ACF differ from normal crypts in size, shape of gland openings, and intensity of methylene blue or indigo carmine staining. The gland lumen shape in ACF depends markedly on histological structure [5]. Moreover, phenotypic and genotypic features of ACF are different from those of normal crypts. They were first described by R. Bird in mice exposed to azoxymethane [6]. ACF are invisible to routine colonoscopy yet are well identifiable in chromoendoscopy. The presence of ACF in humans is identifiable in high-resolution chromoendoscopy with the use of methylene blue or indigo carmine [7,8].

Histopathological features of individual types of ACF have been described and accurately defined by many authors [9].

Main reason of this investigation was comparison of high resolution colonoscopy and standard resolution colonoscopy. Authors wanted to proof that sensitivity of both is on the same level in identity of rectal ACF. Expensive equipment is not common in some regions of Poland

## Material and Methods

Participants provided their written consent to participate in this study. Research was approved by Bioethic Committee at University of Warmia and Mazury in Olsztyn 29.04.2010. (11/2010).

The following were evaluated: duration of chromoendoscopy of the rectal segment examined, type of ACF as well as sensitivity and specificity of the method used to evaluate rectal ACF.

Colonoscopy combined with 0.25% methylene blue staining of the mucosa was performed in 131 patients; their mean age was 60 years (23–85). All examinations were carried out in the Department of General and Minimally Invasive Surgery of the Teaching Hospital, Medical University in Olsztyn. The population study encompassed 73 female and 58 male patients. The mean age was 67 years and 52 years, respectively.

Examinations were performed using a CF Q-165L standard-resolution colonoscope (Olympus), FB-240U biopsy forceps (Olympus) and a “spray” catheter (Olympus).

Prior to examinations, each patient underwent complete colonoscopy. The rectal segment from the pectinate line to the middle rectal fold was stained with 0.25% methylene blue, rinsed with distilled water and assessed for the presence of ACF.

ACF were considered the crypts with darker methylene blue staining, larger in size, of oval or slit-like openings and distinct borders compared to normal crypts. The bioptates were examined by one pathologist.

After staining the mucosa of a given rectal segment, bioptates were collected and divided into 2 groups.

Group 1- bioptates macroscopically defined as ACF
Group 2 –bioptates with macroscopically normal mucosa (control group).

Three hundred and ten bioptates were obtained in group 1 and 357 in group 2. ACF were histologically classified into one of the four groups according to the presence of dysplasia or hyperplasia in accordance with the criteria described earlier.

Bioptates were initially evaluated and oriented under the binocular magnifying glass. The 2- μm tissue sections were prepared using a standard paraffin method and stained with haematoxylin and eosin. Qualitative microscopic histopathological evaluation was carried out under 50-400x magnification. Three hundred and fifty-seven bioptates with macroscopically normal mucosa were assessed in the same way as ACF bioptates.

Once false positive and false negative values were calculated, specificity and sensitivity of endoscopic evaluation were assessed **in comparison with histopathological evaluation.**

In statistical analysis results has shown as numerical data, average, standard deviation, distribution of probability. In order to determine of statistical significance Test U and t-student test were used. Statistica 7.1 and Excel 2007 were used.

## Results

The mean duration of colonoscopy and chromoendoscopy was 26.8 min. In cases where the colon mucosa required additional purification, the time was longer—44.14min.

Differences in times of chromoendoscopy according to the quality of colon preparation were statistically significant, **PV<0.001**.

In the study population, typical ACF were found in 73 patients (p = 0.489), hyperplastic ACF in 49 (p = 0.328), and dysplastic ACF in 16 patients (p = 0.107). Mixed ACF were observed in 11 individuals (p = 0.073).

The study population was divided into three subgroups according to the number of ACF observed, i.e. ACF < 5, 5–10 and >10. ACF < 5 were found in 35 patients (29.41%), 5–10 ACF in 70 (58.82%) and ACF > 10 in 14 individuals (11.76%).

In the population study one type of ACF was observed in 88 patients (p = 0.7394), two types in 29 (p = 0.2437) and three types in 2 individuals (p = 0.0168).

Microscopic confirmations of endoscopic ACF diagnoses (S1 Table). Results found in the control group (S2 Table).

Analysis of data revealed 12 false negative, 2 false positive, 335 truly positive and 298 truly negative values. The sensitivity of the method was found to be 0.96 whereas its specificity 0.99.

## Discussion

Colonoscopy for colon diagnosis supplants the other methods and the demands for its use are increasingly high. Colonoscopy with dyes enables the detection of dysplastic changes, flat neoplastic lesions and ACF. High-resolution chromoendoscopy and endoscopy are essential for ACF diagnosis.

Staining of the entire colon is technically difficult, laborious, time-consuming and arduous for patients [8]. Since the highest mean density of ACF is found in the rectum, the majority of pathologists assessing ACF examine only this segment [5,7,10,11,12,13].

In highly-specialised centres in which chromoendoscopy is routinely used for examinations of the large intestine, the time of complete colonoscopy with staining is 30–60 minutes or even longer. The examination confined to the rectum prolongs the procedure by only 10–15 minutes [7,8,11].

In our material, the mean duration of colonoscopy with staining and rectal ACF evaluation was about 27 minutes, which is not markedly different from the results of other authors [8].

The time consumption of the examination and ACF visualization are significantly affected by proper preparation of the rectum for staining.

The rectal mucosa poorly prepared for staining deteriorates the quality of methylene blue staining of the rectum. Islands of poorly stained mucosa are observed, and ACF evaluation is substantially hindered. Additional rinsing of the rectum also prolongs the examination time. Some authors routinely precede rectal staining with its rinsing with 0.5% solution of glycerin or mucolytic preparation (10–20% N-acetylcysteine) to remove the mucus remains, which in turn enables better penetration of the dye into the mucous membrane of the colon section stained [11,14,15,16]. In our study, the colon was only mechanically purified with polyethylene glycol (Fortrans); no other preparations were used. The method used for staining of our 131 preparations, i.e. 0.25% methylene blue plus rinsing with distilled water, was found sufficient for good identification of rectal ACF. Mucolytics were not used mainly because the surface of ACF-forming colonocytes is less mature and contains low numbers of microvilli compared to the normal surrounding mucosa, which is associated with deficiency of protective mucus.

The concentration of a due and its possible effects on differences in macroscopic evaluation of ACF (borders, intensity of staining) remain open for discussion. The available literature data suggest that the issue is of a minor importance. The researchers evaluating ACF used a variety of concentrations of methylene blue, ranging from 0.01% to 1.0%, and did not determine explicitly the reference concentration for ACF evaluation [11,17,18,12,19,14,15,20]. The concentration of 0.25% used in the present study resulted from our previous experiences and easiness of dilution of methylene blue available in the endoscopic laboratory—0.5% solution. Moreover, it should be remembered that after application of methylene blue onto the mucosal surface, 2–3 minutes are needed for its absorption by the epithelial cells, which is not required when indigo carmine is applied (contrast stain). When this fact is neglected, the identification of ACF is more difficult.

Proper ACF identification should be confirmed by the incidence of their individual types. According to Fenoglio-Preiser, typical and hyperplastic ACF are most common [20]. In our material, these two types were also most frequently observed. Similar incidences of typical and hyperplastic ACF have been reported by other authors [11,13,7,20,21]

The incidences of dysplastic ACF, which are crucial from the carcinogenesis point of view, reported in various studies differ. The lowest percentages of dysplastic ACF were presented by Nascimbeni, Adler, and Rudolph; those reported by Takayama are comparable to our findings [8,11,15,22]. There are also studies in which the percentages in question are higher than those observed in our population. According to Siu et al., the incidence of dysplastic ACF was 73% in the Afro-Americans and 44% in White Americans [21]. A similarly high percentage was reported by Roncucci and co-workers yet their study involved patients with familial adenomatous polyposis (FAP) and sporadic colorectal cancer [23]. Different numbers of dysplastic ACF in comparable groups can result from ethnic, dietary and environmental differences [8,11,15,23]. Similar percentages of various types of ACF in the studies with high-resolution colonoscopes and in our study group evidence that identification of aberrant intestinal crypts using standard-resolution colonoscopes is comparable to that with high-resolution colonoscopes.

Studies on ACF are based on high-resolution colonoscopy using methylene blue or indigo carmine. Standard videoendoscopes are equipped with cameras of 100–300 thousand pixel resolution whereas high-resolution videoendoscopes have cameras of 400 thousand -1 million pixels. In Poland, high-resolution colonoscopy with chromoendoscopy has not been widely used in endoscopic units to identify early lesions; therefore we decided to assess the usefulness of standard-resolution colonoscopes for evaluation of rectal ACF [8,10].

ACF as the lesions of higher mitotic activity absorb higher amounts of a stain than the adjacent, healthy tissue, which makes them well visible and circumscribed from the surrounding tissues. This effect is clearly visible even when standard endoscopes are used without magnification and digital enhancement of the image.

During macroscopic qualification of ACF, it is essential to follow the principles described by Takayame, Pretlow, Roncucci [11,18,23]. In most cases, ACF are the changes slightly elevated above the normal rectal mucosa surrounding them and distinctly depressed. Unlike high-resolution colonoscopes, standard–resolution devices do not allow to evaluate the luminal patterns, described by Roncucci et al.[23].

The concurrence of endoscopic and histopathological images constitutes a clinical problem. According to Rasheed and colleagues, histopathologists confirm only slightly more than 50% of endoscopy-identified ACF. About 5% of bioptates assessed endoscopically as normal are described as ACF by pathologists [24]. What do these discrepancies result from and what are they affected by?

The differences between evaluations performed by endoscopists and pathologists are likely to be associated with the lack of 100% features of macroscopic changes suggestive of ACF; some criteria described by Roncucci and co-workers can be identical as those in a small hyperplastic polyp [23]. Considering the above, Rasheed states that endoscopic ACF criteria should fulfill certain conditions and the distal colon segment to be stained should be well defined[24]. Despite such assumptions, 100% macroscopic changes for reliable ACF identifications have not been determined. The discrepancies mentioned above can also be affected by the learning curve of ACF of both pathologists and endoscopists [25].

According to some other authors, the consistency of endoscopic and histopathological examinations ranges from 53% to 92% [8,11].

In our population, the sensitivity of chromoendoscopy with a standard-resolution colonoscope was 96% while its specificity was 99%. Similar percentages were reported by Schoen et al, i.e. 92% of consistency between endoscopic and pathological findings [25]. In their study, endoscopic examinations were carried out by two independent and suitably trained pathologists; moreover, chromoendoscopy was also performed by appropriately trained endoscopists.

What do the discrepancies in findings reported by the trained personnel result from?

Firstly, ACF can be composed of only few-several crypts and the material for histopathological evaluation can be improperly collected with biopsy forceps. Bioptates can contain no pathological tissue or only its small fragment. Furthermore, even slight bleeding during biopsy can hinder proper evaluation of ACF 925.

The oblique positioning of biopsy forceps or improper plane of cutting of paraffin blocks can also impair microscopic assessment of biopsy preparations.

High sensitivity and specificity in our population is likely to result from a small size of the group, lower probability of errors and collection of bioptates only from the lesions, which were undoubtedly ACF. Our initial study and its findings encourage to further research.

Since standard-resolution chromoendoscopy enables the evaluation of ACF in the time similar to that of high-resolution chromoendoscopy and indentifies the types of ACF comparably to more technologically advanced devices, is its reliability comparable to that of histopathologic examinations? Our findings seem to suggest that it is.

Analysis of literature data from the centres experienced in ACF evaluation reveals that proper preparation of the intestine, endoscopic and histopathological trainings in well-prepared centres as well as suitable endoscopic and histological criteria of ACF evaluation are essential for proper endoscopic and histopathological identification of ACF.

## Conclusions

Identification of rectal ACF using standard-resolution colonoscopy combined with rectal mucosa staining with 0.25% methylene blue is characterised by high sensitivity and specificity.

## Supporting Information

S1 Fig Typical ACF(arrow)[the author's own material]. (DOCX)

S2 Fig Mixed ACF(arrow)[the author's own material]. (DOCX)

S3 Fig Dysplastic ACF (arrow)[the author's own material]. (DOCX)

S4 Fig Hyperplastic ACF (arrow)[the author's own material]. (DOCX)

S1 Table Microscopic confirmations of endoscopic ACF diagnoses. (DOCX)

S2 Table Results found in the control group. (DOCX)
