# Supplementary figures and images for: Chromoendoscopy with a Standard-Resolution Colonoscope for Evaluation of Rectal Aberrant Crypt Foci
Source: PLoS One. 2016 Feb 17;11(2):e0148286. doi: 10.1371/journal.pone.0148286 (PMC4757420; doi:10.1371/journal.pone.0148286)

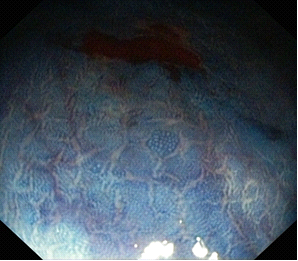


S 1 Fig. Typical ACF(arrow)[the author's own material]

Supplement: S1 Fig — (DOCX) [file pone.0148286.s001.docx]

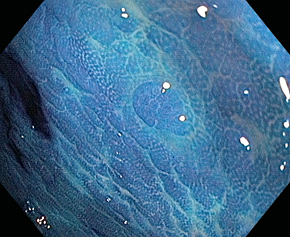


S 2 Fig. Mixed ACF(arrow)[the author's own material]

Supplement: S2 Fig — (DOCX) [file pone.0148286.s002.docx]

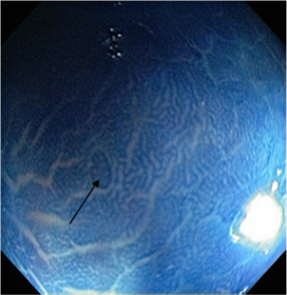


S 3 Fig. Dysplastic ACF (arrow)[the author's own material]

Supplement: S3 Fig — (DOCX) [file pone.0148286.s003.docx]

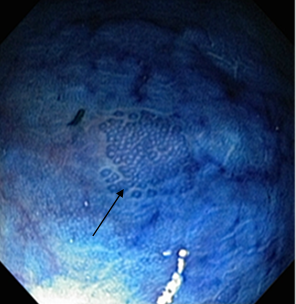


S 4 Fig. Hyperplastic ACF (arrow)[the author's own material]

Supplement: S4 Fig — (DOCX) [file pone.0148286.s004.docx]
