# Supplementary material for: Chromoendoscopy with a Standard-Resolution Colonoscope for Evaluation of Rectal Aberrant Crypt Foci
Source: PLoS One. 2016 Feb 17;11(2):e0148286. doi: 10.1371/journal.pone.0148286 (PMC4757420; doi:10.1371/journal.pone.0148286)
Supplement: S1 Table — (DOCX) [file pone.0148286.s005.docx]

S1 Table. Microscopic confirmations of endoscopic ACF diagnoses

| Number of bioptates | ACF <5 | Total number of bioptates in the group | False negative value | Accuracy of endoscopic diagnosis |
| --- | --- | --- | --- | --- |
| 1 bioptate | 17 | 17 | 0 | 100% |
| 2 bioptates | 13 | 26 | 2 | 92.31% |
| 3 bioptates | 5 | 15 | 1 | 93.33% |
|  | 5-10 ACF |  |  |  |
| 3 bioptates | 70 | 210 | 8 | 96.20% |
|  | ACF>10 |  |  |  |
| 3 bioptates | 14 | 42 | 1 | 97.62% |
