# Supplementary material for: Chromoendoscopy with a Standard-Resolution Colonoscope for Evaluation of Rectal Aberrant Crypt Foci
Source: PLoS One. 2016 Feb 17;11(2):e0148286. doi: 10.1371/journal.pone.0148286 (PMC4757420; doi:10.1371/journal.pone.0148286)
Supplement: S2 Table — (DOCX) [file pone.0148286.s006.docx]

S2 Table. Results found in the control group

| Number of bioptates | Total number of bioptates in the group | False positive value | Accuracy of endoscopic diagnosis |
| --- | --- | --- | --- |
| 3 | 357 | 2 | 99.44% |
